# Platelet-rich plasma improves cyclophosphamide-induced interstitial cystitis in rat models through the toll-like receptor 4/nuclear factor-kappa B signalling pathway

**DOI:** 10.1007/s10157-025-02660-5

**Published:** 2025-03-25

**Authors:** Yufan Wu, Lei Chen, Minzhe Xu, Linya Yao, Shiyao Yang, Xiaojie Ang, Weiguo Chen

**Affiliations:** 1 Department of Urology, The First Affiliated Hospital of Soochow University, 188 Shizi Street, Gusu District, Suzhou, 215000 JiangSu Province China; 2 Department of Urology, Kunshan Hospital of Traditional Chinese Medicine, Kunshan, JiangSu Province China; 3 Department of Urology, Kunshan Sixth People’s Hospital, Kunshan, JiangSu Province China; 4 Department of Orthopedics, Affiliated Kunshan Hospital of Jiangsu University, Suzhou, JiangSu Province China; 5 Department of Urology, The 901 Hospital of Chinese People’S Liberation Army Joint Service Support Unit, No. 424 Changjiang West Road, Shushan District, Hefei, 230031 AnHui Province China

**Keywords:** Interstitial cystitis, Platelet-rich plasma, Cyclophosphamide, Female SD rats, Kappa B signalling pathways

## Abstract

**Objective:**

To investigate the therapeutic effect of platelet-rich plasma (PRP) on a cyclophosphamide (CYP)-induced interstitial cystitis (IC) rat model.

**Methods:**

A CYP-induced IC rat model (75 mg/kg every 3 days, with a total of five injections) was used to evaluate the therapeutic effects of PRP. Here, PRP was administered via bladder irrigation (every 2 days, with a total of three irrigations), and bladder tissue was analysed for inflammation and histological changes. The toll-like receptor 4 (TLR4)/nuclear factor-kappa B (NF-κB) signalling pathway was assessed using real-time quantitative polymerase chain reaction and ribonucleic acid sequencing. In addition, lipopolysaccharide (LPS)-induced SV-HUC-1 cells (10 μg/LPS and 2.5 mM adenosine triphosphate) were employed to investigate the inflammatory response and the effects of PRP on the TLR4/NF-κB signalling pathway.

**Results:**

The PRP treatment significantly improved the bladder tissue condition in the CYP-induced IC rat model, as evidenced by reduced inflammation and histological damage. The damage and shedding of the superficial epithelium of the bladder mucosa were notably decreased following PRP bladder instillation. Importantly, the expression of ZO-1, a key marker of epithelial integrity, was upregulated in PRP-treated rats, indicating enhanced bladder epithelial function. High-throughput analysis revealed that PRP alleviated bladder mucosal injury in the IC rat model through the TLR4/NF-κB signalling pathway. In LPS-induced SV-HUC-1 cells, PRP treatment also increased ZO-1 expression, decreased CDH1 expression and regulated the TLR4/NF-κB signalling pathway.

**Conclusion:**

Platelet-rich plasma treatment may improve the expression of ZO-1 and CDH1 in urinary epithelium in vitro by mediating the TLR4/NF-κB pathway, which is effective in the treatment of IC.

## Introduction

Interstitial cystitis (IC) is a chronic inflammatory disease characterised by persistent bladder pain and frequent urination, severely affecting patients’ quality of life [1]. Although the specific cause of IC remains unclear, studies suggest that damage to the bladder mucosal barrier and local inflammatory responses play a crucial role in the pathogenesis of the disease [2]. Currently, treatment options for IC are limited and often fail to achieve desirable effects, making it necessary to explore new therapeutic approaches.

Autologous platelet-rich plasma (PRP) is the processed liquid fraction of autologous peripheral blood with a platelet concentration above the baseline [3]. Platelet-rich plasma therapies have been used for various indications for more than 30 years, resulting in considerable interest in the potential of autologous PRP in regenerative medicine. The use of PRP in tissue regeneration is a rapidly evolving area for both clinicians and researchers and is being employed in various fields, including osteoarthritis [4], rotator cuff repair [5], and bone regeneration [6]. Platelet-rich plasma therapy is based on the fact that platelet growth factors support the three phases of wound healing and repair cascade (inflammation, proliferation, remodelling). Many different PRP formulations have been evaluated, originating from human, in vitro and animal studies. However, recommendations from in vitro and animal research often lead to different clinical outcomes because it is difficult to translate non-clinical study outcomes and methodology recommendations to human clinical treatment protocols. In recent years, progress has been made in understanding PRP technology and the concepts for bioformulation, and new research directives and new indications have been suggested [7]. As an emerging biologic treatment, PRP has attracted attention for its rich content of growth factors and potential to promote tissue repair [8], demonstrating efficacy in enhancing wound healing, reducing inflammation and alleviating pain across various fields [9–11]. However, the application and mechanisms of PRP in the treatment of IC require further investigation.

In addition, studies have shown that the toll-like receptor 4 (TLR4) and nuclear factor-kappa B (NF-κB) signalling pathways play central roles in the pathological processes of many inflammatory diseases. Their activation promotes the release of inflammatory cytokines, exacerbating tissue damage [12, 13]. Furthermore, the TLR4/NF-κB signalling pathway has emerged as a crucial therapeutic target in the treatment of bladder inflammation [14]. Studies have demonstrated that either genetic deletion or pharmacological inhibition of TLR4 can effectively prevent bladder inflammation in mouse models of cyclophosphamide (CYP)-induced cystitis [15, 16]. This evidence supports the involvement of TLR4/NF-κB signalling in the pathogenesis of CYP-induced IC, highlighting its potential as a key modulator in the inflammatory response associated with cystitis.

Given this, we hypothesise that PRP may exert therapeutic effects on IC by modulating the TLR4/NF-κB signalling pathway. The aim of this study is to explore the therapeutic effects of PRP in a rat model of IC induced by CYP, specifically analysing its impact on the TLR4/NF-κB signalling pathway and further revealing the underlying mechanisms through which PRP improves IC.

## Materials and methods

### Experimental reagents and instruments

The following reagents and instruments were obtained: CYP (B0150, Yuchun Bio, Shanghai, China); haematoxylin and eosin Toluidine blue stain; Masson’s trichrome stain; PureLink ribonucleic acid (RNA) (Ambion, Austin, Texas, USA); Ezol Reagent (B002-v001, GenePharma, Shanghai, China); GoScript RT system (A5001, Promega, Madison, WI, USA); GoTaq qPCR Master Mix (A6001, Promega, Madison, WI, USA); qRT-PCR Plus System (Stratagene, La Jolla, CA, USA); Mouse anti-TLR4 (66350-1-Ig; Proteintech, CHI, USA); Rabbit anti-ZO-1 (AF5145; Affinity, OU, USA); Rabbit anti-E-cadherin (ab40772; Abcam, Cambridge, UK); Rabbit anti-NF-kB p65 (10745-1-AP; Proteintech, CHI, USA); primary and secondary antibodies and washing solutions (ZO-1, E-Cadherin, TLR4, NF-kB p65, ABcam, Cambridge, UK); DMEM/F-12 K medium (31800–105, Gibco, USA); cell counting kit-8 (CCK-8) (GW770, Dojindo, Tokyo, Japan); a microplate reader (Multiskan SkyHigh, Thermo Fisher Scientific); Cell-Light^TM^ EdU Apollo567 In vitro Kit (RiboBio, Guangzhou, China); a flow cytometer (BD Biosciences); CycleTEST™ PLUS DNA Reagent Kit (BD Biosciences); pentobarbital sodium (Jiangsu Hengfengqiang Biotechnology Co., Ltd.); an F3 catheter (150301J, Shanghai Shangyi Kangge Medical Equipment Co., Ltd.); a 15-channel physiological recorder (MP150-WSW, BIOPAC, USA); a 5% CO_2_ incubator (XD-101, SANYO, Japan); and a – 80 ℃ refrigerator.

### Preparation of platelet-rich plasma

The preparation of PRP was conducted following the protocol described by Nagae et al. [17]. Due to the rapid coagulation and slow filling rate associated with superficial veins, blood extraction was performed via the intracardiac route of rats under sedation. Approximately 4.5 mL (range: 3.5–6.0 mL) of blood was promptly transferred into tubes containing 3.2% citrate. Pre-instillation blood samples were collected and immediately transported to the laboratory for PRP preparation. Initial centrifugation was performed at 1,600 rpm for 15 min to separate the supernatant and buffy coat. Subsequent centrifugation of the remaining plasma at 2,800 rpm for 8 min facilitated platelet precipitation. The platelet-reduced supernatant was discarded, yielding an average of 1 mL (range: 0.8–1.2 mL) of PRP from the buffy coat.

### Interstitial cystitis rat model

In this study, 40 specific pathogen-free grade healthy female SD rats (180–200 g in weight) were purchased from Qinglongshan Experimental Animal Center, Nanjing, China. The rats were housed in standard cages at a constant temperature (22 ℃±1 ℃) and humidity (a relative humidity of 60%±5%), with free access to food and water, under a 12-h light/dark cycle. After 1 week of adaptive feeding, animal modelling and medicated administration were conducted. The SD rats were randomly divided into four groups: the control group (no model and phosphate buffered saline [PBS] intraperitoneal injection), the PBS group (PBS intraperitoneal injection), the CYP group (IC model constructed through CYP intraperitoneal injection), and the CYP+PRP group (model and PRP bladder irrigation). Here, CYP was injected intraperitoneally at a dose of 75 mg/kg every 3 days, with a total of five injections. On the 15th day, 0.3 ml of PRP was irrigated into the bladder every 2 days at a dose of 2 ml/time, for a total of three bladder irrigations. On the 21st day, following 10% chloral hydrate anaesthesia by intraperitoneal injection, the bladder tissue was removed, weighed and fixed, and stored in the refrigerator at – 80 ℃. The body weight and wet bladder weight of each rat of each group were measured using a precision balance.

## Analyses

### Ribonucleic acid sequencing analyses

Ribonucleic acid was extracted from rat bladder tissue using a standard homogenisation and purification protocol. Briefly, tissue samples were transferred into pre-chilled RNase-free tubes on ice and lysis buffer containing 2-mercaptoethanol was added. The samples were homogenised using a rotor-stator homogeniser for 30–45 s, depending on tissue weight, and then centrifuged at approximately 2,600 × g for 5 min. The supernatant was collected, and 1 volume of 70% ethanol was added to the lysate, followed by vortexing to disperse any precipitate. The sample was then transferred to a spin cartridge, centrifuged at 12,000 × g for 15 s and the flow-through discarded. This process was repeated until the entire sample was processed. Following this, RNA was washed with Wash Buffer I and Wash Buffer II, with centrifugation at 12,000 × g for 15 s at room temperature between steps. After washing, RNA was eluted by adding 30 μL (3 × 100 μL) of RNase-free water, incubating for 1 min and centrifuging at 12,000×g for 2 min; RNA yield and quality were assessed using UV absorbance at 260 nm and the Quant-iT™ RiboGreen™ RNA Assay Kit. Then complementary deoxyribonucleic acid (cDNA) synthesis was performed, and the libraries were sequenced using the Illumina NovaSeq 6000 system (Illumina; paired-end, 150 bp).

### Ribonucleic acid extraction and real-time quantitative polymerase chain reaction assays

The total RNA of the bladder tissue was extracted using the Ezol reagent method. The target cDNA was synthesised through reverse transcription (RT) using random primers and the GoScript RT system. A real-time quantitative polymerase chain reaction (qRT-PCR) was performed using the GoTaq qPCR Master Mix and qRT-PCR Plus System. A two-tailed Student’s *t* test or one-way analysis of variance (ANOVA) was used for statistical analysis. The sequences of the Tjp1, Tnf, Tlr4, Nfkb1, Nfkb2, Tlr3, Il4, Il6, Tgfb1, ZO-1, CDH1, TLR4 and NF-κB1 primers are shown in Table 1.

**Table1:** The sequences of theTjp1, Tnf, Tlr4, Nfkb1, Nfkb2, Tlr3, Il4, Il6, Tgfb1, ZO1, CDH1, TLR4 and NFKB1 primers

	Forward	Reverse
Tjp1	5ʹ GAGCTACGCTTGCCACACTGT 3ʹ	5ʹ TCGGATCTCCAGGAAGACACTT 3ʹ
Tnf	5ʹ CACCATGAGCACGGAAAGCA 3ʹ	5ʹ GCAATGACTCCAAAGTAGACC 3ʹ
Tlr4	5ʹ TGGCATCATCTTCATTGTCC 3ʹ	5ʹ CAGAGCATTGTCCTCCC 3ʹ
Nfkb1	5ʹ TTCAACATGGCAGACGACGA 3ʹ	5ʹ AGGTATGGGCCATCTGTTGAC 3ʹ
Nfkb2	5ʹ TACAAGCTGGCTGGTGGGGA 3ʹ	5ʹ GTCGCGGGTCTCAGGACCTT 3ʹ
Tlr3	5ʹ GATTGGCAAGTTATTCGTC 3ʹ	5ʹ GCGGAGGCTGTTGTAGG 3ʹ
Il4	5ʹ CGTGATGTACCTCCGTGCTT 3ʹ	5ʹ GTGAGTTCAGACCGCTGACA 3ʹ
Il6	5ʹ CAGAGGATACCACCCACAACAGA 3ʹ	5ʹ CAGTGCATCATCGCTGTTCATACA 3ʹ
Tgfb1	5ʹ GCAACAACGCAATCTATGAC 3ʹ	5ʹ CCTGTATTCCGTCTCCTT 3ʹ
ZO1	5ʹ GCTAAGAGCACAGCAATGGA 3ʹ	5ʹ GCATGTTCAACGTTATCCAT 3ʹ
CDH1	5ʹ AGAACGCATTGCCACATACAC 3ʹ	5ʹ GAGGATGGTGTAAGCGATGG 3ʹ
TLR4	5ʹ GGGTGAGAAACGAGCT 3ʹ	5ʹ TTGTCCTCCCACTCGA 3ʹ
NFKB1	5ʹ GGCAGCACTACTTCTTGACC 3ʹ	5ʹ CAGCAAACATGGCAGGCTAT 3ʹ
GAPDH	5ʹ ATCACCATCTTCCAGGAGCG 3ʹ	5ʹ CAAATGAGCCCCAGCCTTC 3ʹ

### Immunofluorescence staining

Mouse bladder tissues were immersed in the OCT compound and frozen immediately using liquid nitrogen. The frozen tissue blocks were sectioned using a cryostat into 3-µm slices. The tissue sections were placed in a heater for 1 h, followed by three PBS washes for 10 min each. The sections were then covered by a solution containing 0% goat serum/2% bovine serum albumin/0.2% Triton X-100 and incubated at room temperature for 1 h. Then the tissue sections were incubated overnight at 4℃ with any of the four different primary antibodies depending on the experiment: mouse anti-TLR4 (1:200), rabbit anti-ZO-1 (1:500), rabbit anti-E-cadherin (1:500) and rabbit anti-NF-kB p65 (1:200). After staining with the primary antibody (1:500), the tissue sections were incubated in a secondary antibody for 1 h at room temperature.

### Western blot analysis

The extracted bladder protein was loaded into 10% SDS-polyacrylamide gel and transferred onto a polyvinylidene fluoride membrane following electrophoresis. Then the blots were incubated overnight at 4 ℃ with any of the four different primary antibodies: mouse anti-TLR4 (1:500), rabbit anti-ZO-1 (1:1,000), rabbit anti-E-cadherin (1:1,000), rabbit anti-NF-κB p65 (1:500) and mouse anti-GAPDH (1:1,000). After staining with the primary antibodies (1:2,000) and washing, the ECL Plus assay kit was used for colour rendering.

### Modelling and administering of SV-HUC-1 cells

The SV-HUC-1 cells used in this study were obtained from Nanjing University of Traditional Chinese Medicine. The cells were cultured in a 37 ℃, 5% CO_2_ incubator with DMEM/F-12 K medium containing 10% foetal calf serum, 100 U/mL of penicillin and 100 U/mL of streptomycin. Then 10 μg/L of lipopolysaccharide (LPS) and 2.5 mM of adenosine triphosphate (ATP) were added to stimulate the cells for 12 h. The administration was divided into three groups: the control group, the model group (10 μg/LPS and 2.5 mM ATP) and the LPS+PRP group (10 μg/LPS and 2.5 mM ATP and 1 ml PRP treatment).

### Cell counting kit-8 assay

Cell proliferation rates were measured using a CCK-8 assay. The different groups of SV-HUC-1 cells (5×10^3^ cells/well) were seeded in a 96-well plate. After 0, 24, 48 and 72 h of culture, 10 μL of CCK-8 solution was added, after which the cells were incubated for 2 h at 37 ℃. The absorbance was measured in a microplate reader (Multiskan SkyHigh, Thermo Fisher Scientific) at 450 nm.

### Ethynyl deoxyuridine analysis

The proliferative capacity of different groups of SV-HUC-1 cells was analysed using the Cell-Light^TM^ EdU Apollo567 In Vitro Kit. Transfected cells were incubated with 5 μM of ethynyl deoxyuridine (EdU) according to the manufacturer’s instructions. Thereafter, the cells were fixed in 4% formaldehyde for 30 min and permeabilised with 0.5% Triton-X100 for 10 min. Here, EdU-positive cells were examined under a fluorescence microscope in dark conditions.

### Flow cytometric analysis

The different groups of SV-HUC-1 cells were stained with fluorescein isothiocyanate-Annexin V and propidium iodide and analysed via flow cytometry. Transfected cells were stained using the CycleTEST™ PLUS DNA Reagent Kit according to the manufacturer’s instructions. To examine the cell cycle, cells were analysed via flow cytometry. The relative number of cells in G0/G1, S and G2/M phases was determined and compared between the groups.

### Statistical analysis

Statistical analyses were conducted using SPSS 19.0 software (IBM, Armonk, NY, USA). A two-tailed Student’s *t* test was performed for the comparison between two groups. One-way ANOVA was performed for comparisons between three or more groups based on one factor. The CCK-8 results were analysed using repeated measures ANOVA, with P values of <0.05 considered statistically significant.

## Results

### Construction of the interstitial cystitis model using cyclophosphamide

**Fig. 1:**
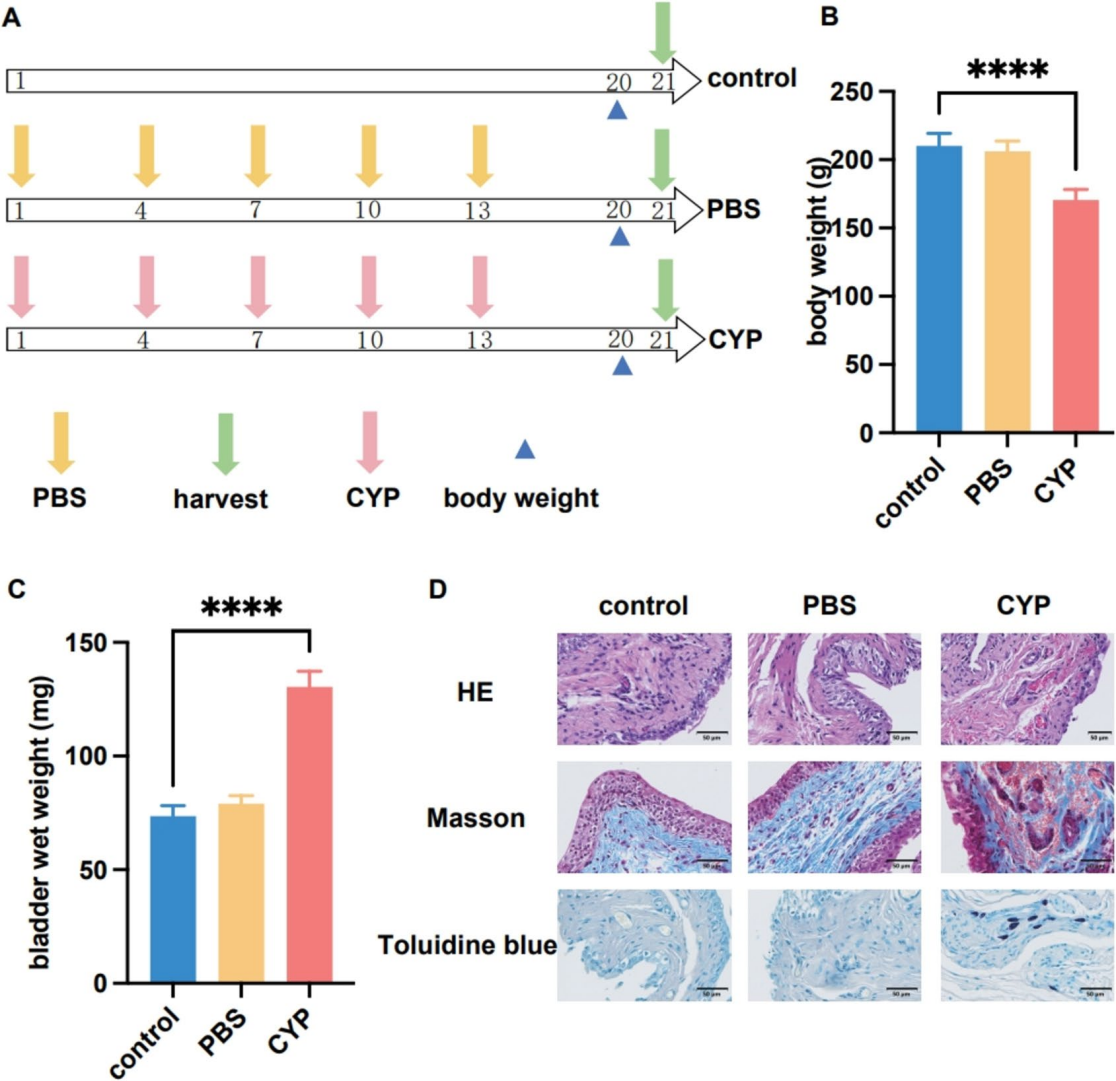
Construction of an interstitial cystitis model by CYP. **A** Modelling process for interstitial cystitis in rats. **B** The body weight between different groups. **C** The wet weight of the bladder between different groups. **D** The different histological staining methods between the CYP model group the control and the PBS group. ****P < 0.0001

In this study, a rat IC model experiment (Fig. 1A) was conducted and divided into the control group, the PBS group and the CYP group. The PBS group and CYP group were injected with the same volume of PBS or CYP solution (0.6 mL) during the experiment. After 20 days of the experiment, the body weight and wet bladder weight of the rats were measured. Results showed that the CYP model group experienced weight loss, with a significant reduction in body weight compared with the control and PBS groups (*P* < 0.0001) (Fig. 1B). Conversely, the wet weight of the bladder in the CYP model group significantly increased, far exceeding that of the control and PBS groups (*P* < 0.0001) (Fig. 1C). Histological staining also confirmed the reliability of the model. Through different histological staining methods, significant differences were observed between the CYP model group and the control group and the PBS group (Fig. 1D), indicating that bladder tissue had damage and inflammation.

### Effects of platelet-rich plasma on the cyclophosphamide-induced interstitial cystitis model

**Fig. 2:**
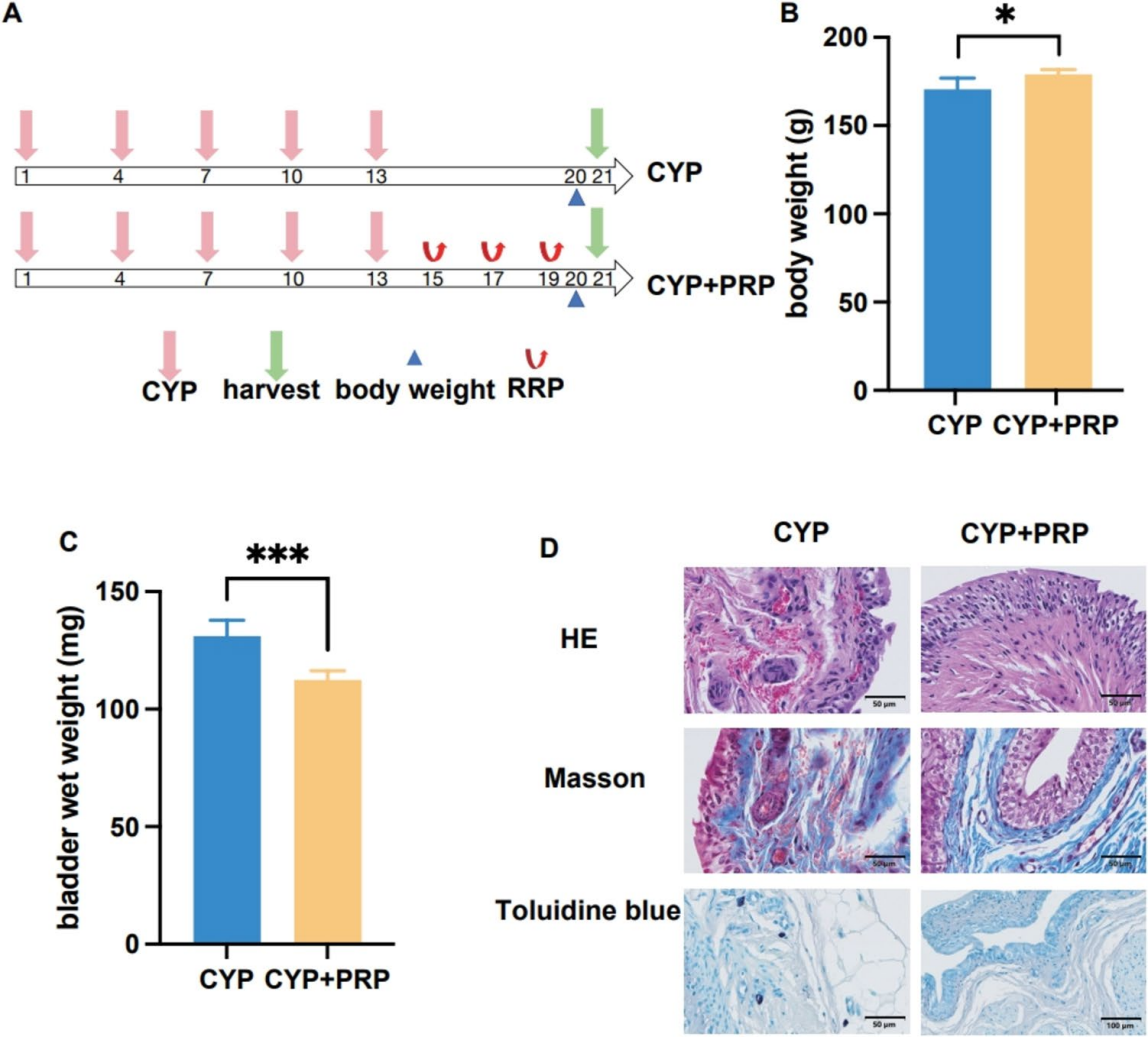
Effects of platelet-rich plasma on the interstitial cystitis model by CYP. **A** The treatment process for interstitial cystitis in rats. **B** The body weight between different groups. **C** The wet weight of the bladder between different groups. **D** The different histological staining methods between the CYP model group and the treatment group. *P < 0.05, ***P < 0.001

Following intraperitoneal injection of CYP, rats were treated with PRP bladder instillation (Fig. 2A). The body weight of each group was measured on the 20th day, and the bladder tissue was collected on the 21st day to measure the wet weight of the bladder tissue. The body weight of the rats in the PRP treatment group increased compared with the untreated group (*P *< 0.05) (Fig. 2B), and the wet bladder weight significantly decreased, possibly indicating a reduction in bladder tissue oedema and inflammation (*P *< 0.001) (Fig. 2C). Histological staining results showed that PRP promoted the recovery of bladder epithelial integrity and reduced the inflammatory response. The number of dark-blue-stained mast cells in the submucosal layer and around the blood vessels of the bladder significantly decreased following PRP treatment (Fig. 2D).

### Differentiation expression analysis of toll-like receptor signalling pathway between cyclophosphamide and cyclophosphamide+platelet-rich plasma group via high-throughput analysis

**Fig. 3:**
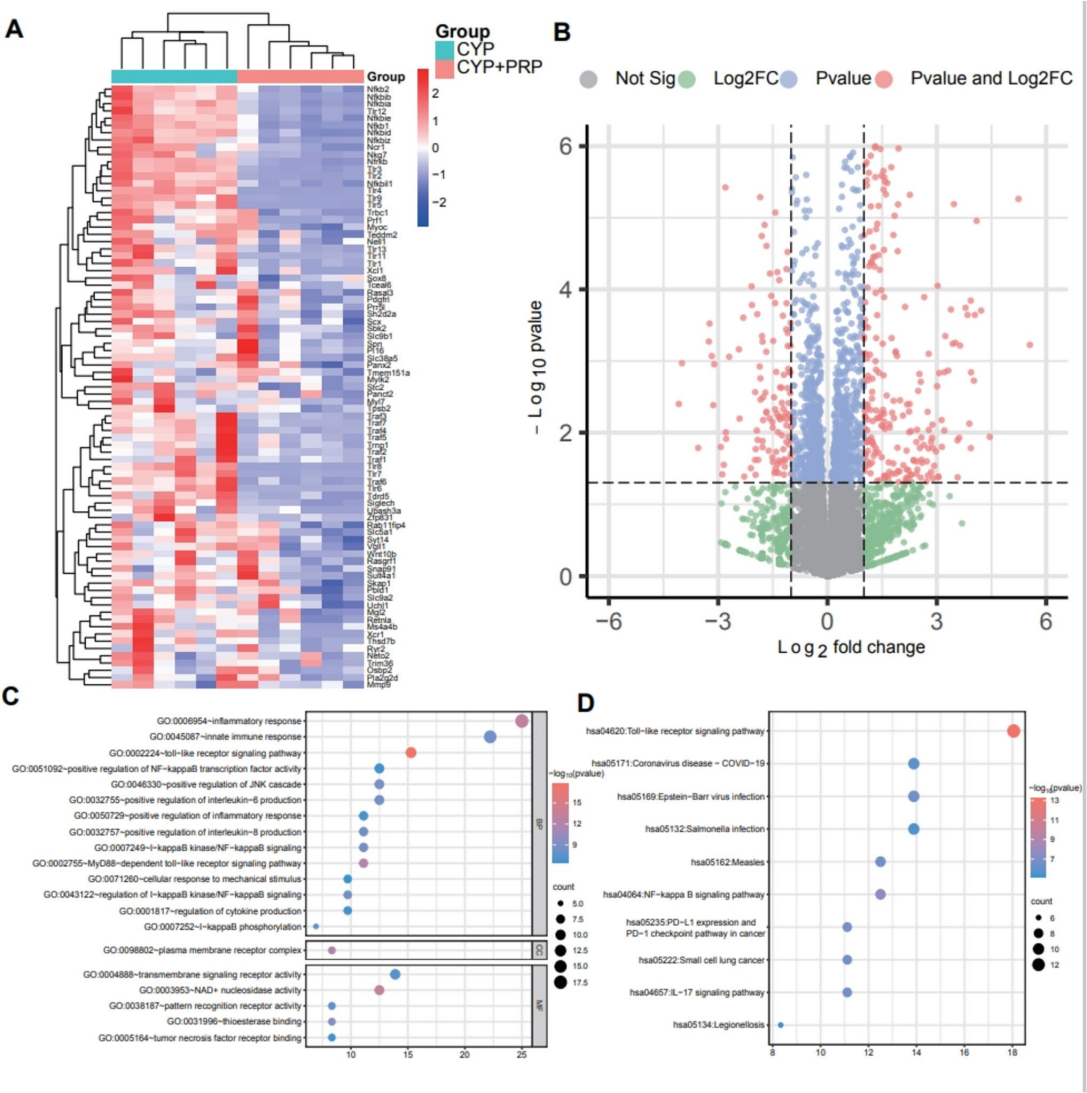
High-throughput analysis between CYP and CYP+PRP group. **A** The heatmap of the high-throughput RNA sequencing between six pairs of samples (CYP group and CYP+PRP group). **B** The volcano plot of the high-throughput RNA sequencing between six pairs of samples (CYP group and CYP+PRP group, fold changes > 1.0, P values < 0.05). **C** The GO analyses of the downregulated RNAs after treatment. **D** The KEGG analyses of the downregulated RNAs after treatment

Following RNA sequencing analysis of CYP group and CYP+PRP group samples, multiple RNA expression differences were found. As shown in the heatmap (Fig. 3A) and volcano plot (Fig. 3B), high-throughput RNA sequencing identified 102 upregulated RNAs and 83 downregulated RNAs (fold changes: >1.0, P values: < 0.05). Subsequently, Kyoto Encyclopedia of Genes and Genomes and gene oncology analyses were conducted on the significantly downregulated RNAs following treatment, indicating that these RNAs were mainly enriched in inflammatory responses, immune responses and pathways involving toll-like receptors and the NF-κB signalling pathway (Fig. 3C and 3D).

### Improvement of bladder mucosal damage in interstitial cystitis rat model through the toll-like receptor 4/nuclear factor-kappa B signalling pathway

**Fig. 4:**
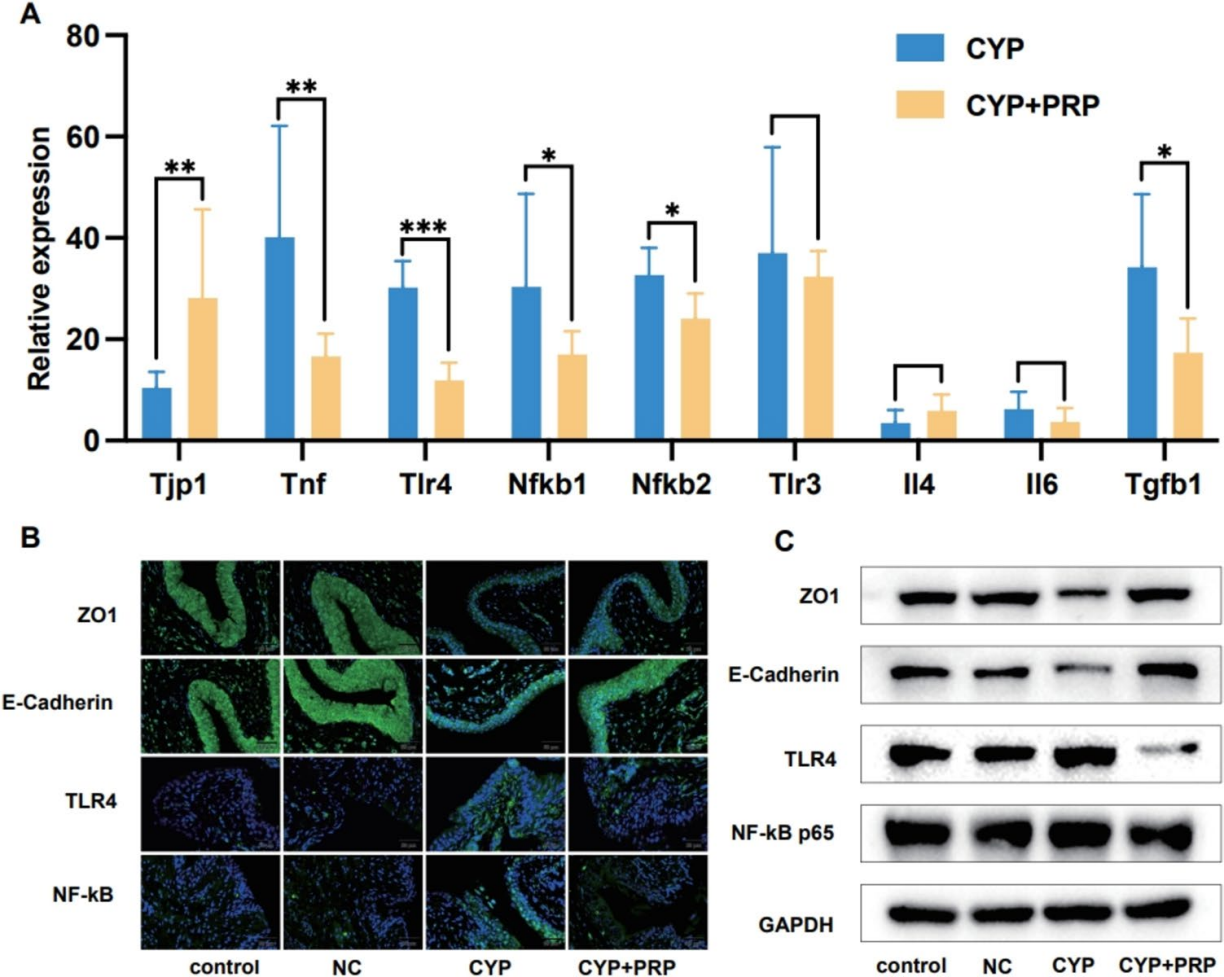
The validation of the different expressed genes by PCR, WB and immunofluorescence staining. **A** The PCR validation on some genes with significant differences in the sequencing results (Tjp1, Tnf, Tlr4, Nfkb1, Nfkb2, Tlr3, Il4, Il6, Tgfb1. PCR). **B** The expression levels of TLR4, NF-κB, ZO1 and E-Cadherin at the histological levels. **C** The expression levels of TLR4, NF-κB, ZO1 and E-Cadherin at the protein levels. *P < 0.05, **P < 0.01, ***P < 0.001

The RNA sequencing results were verified via PCR detection. It was found that the expression of characteristic genes in the bladder epithelium of the PRP treatment group was upregulated and the expression of inflammation-related RNA was downregulated (Fig. 4A). At the same time, the expression levels of TLR4 and NF-κB were measured at the histological and protein levels, as well as the expression levels of the urinary epithelial-specific molecules, ZO-1 and E-cadherin (Fig. 4B and 4C). It was found that in the CYP group, the expression levels of the ZO-1 and E-cadherin molecules were significantly lower than those in the control group, indicating urinary epithelial damage in the CYP model, whereas the expression levels of TLR4 and NF-κB were significantly higher. The improvement effect of PRP on bladder mucosa was, therefore, further verified.

### Improvement in the proliferation of lipopolysaccharide-induced SV-HUC-1 cells by platelet-rich plasma in vitro through the toll-like receptor 4/nuclear factor-kappa B signalling pathway

**Fig. 5:**
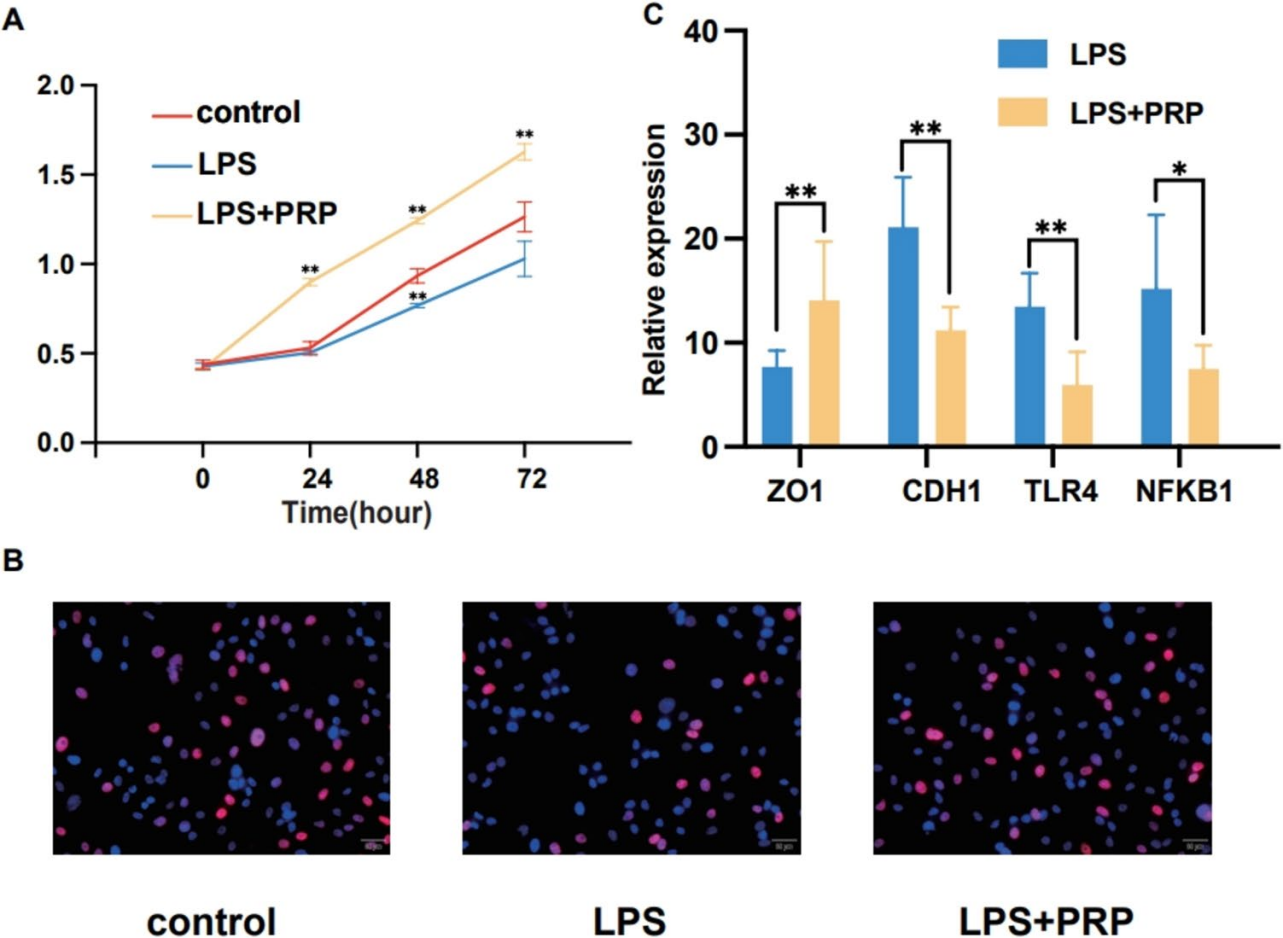
The proliferation of LPS-induced SV-HUC-1 was improved by PRP in vitro through TLR4/NF-κB signalling pathway. **A** The cell proliferation levels were assessed by CCK-8 assays. **B** The cell proliferation levels were assessed by EdU assays. **C** The PCR validation on ZO1, CDH1, TLR4 and NFKB1 between different groups. *P < 0.05, **P < 0.01

In vitro experiments further validated the effects of PRP. Here, SV-HUC-1 cells pre-treated with LPS underwent PRP treatment, and their cell proliferation levels were assessed in comparison with those of the blank and model groups using CCK-8 and EdU assays (Fig. 5A and 5B). It was found that the proliferation activity of SV-HUC-1 cells pre-treated with LPS was significantly reduced but partially recovered following PRP treatment. In addition, the differential expression of ZO-1, CDH1, TLR4 and NF-κB at the RNA level between the model and treatment groups was verified, suggesting that PRP might improve the expression of urinary epithelial ZO-1 and CDH1 in vitro by mediating the TLR4/NF-κB pathway (Fig. 5C).

## Discussion

In recent years, the treatment of IC has become a challenging issue due to the disease’s chronic inflammatory nature and the limited treatment options. The purpose of this study was to investigate the therapeutic effect of PRP on a CYP-induced IC rat model and to explore its effect on the TLR4/NF-κB signalling pathway, so as to elucidate the potential mechanism of PRP improving IC.

As a biological treatment method, PRP is rich in growth factors and cytokines, which has a significant effect on tissue repair and regeneration [17]. Studies have shown that PRP can promote wound healing, inhibit inflammatory response and show potential in the treatment of various diseases [18]. Consistent with previous studies, the results of the present study showed that following PRP treatment, the weight of IC rats increased, the wet bladder weight decreased significantly and the bladder mucosa damage was alleviated, which was consistent with the mechanism of PRP in tissue repair. This suggests that PRP may act as a therapeutic intervention for IC by promoting tissue repair and reducing inflammation.

The TLR4/NF-κB signalling pathway is considered to play an important role in the pathogenesis of IC. The activation of TLR4 and NF-κB can promote inflammatory response and tissue damage [19]. The present study found that PRP treatment could upregulate the expression of bladder epithelial characteristic genes, downregulate the expression of inflammation-related RNA and inhibit the activation of the TLR4/NF-κB signalling pathway, which verified the mechanism of PRP improving IC through this pathway. This is consistent with the results of previous studies, indicating that PRP may play a therapeutic role by affecting the TLR4/NF-κB signalling pathway [20]. In addition, the results of high-throughput RNA sequencing and PCR validation in this study further support the therapeutic effect of PRP on IC inflammation damage through the TLR4/NF-κB signalling pathway. These findings are consistent with existing literature that highlights the important role of the TLR4/NF-κB signalling pathway in various inflammatory diseases [21–24], where its activation leads to the release of inflammatory cytokines and the exacerbation of tissue damage. These results again suggest that PRP may act as a therapeutic intervention for IC by promoting tissue repair and reducing inflammation.

In addition, RNA sequencing analysis showed that multiple RNAs were significantly downregulated following PRP treatment, mainly involving inflammatory response, immune response and related signalling pathways. Studies have shown that PRP treatment has a significant effect on inhibiting inflammatory response [25]. In summary, the results of this study show that PRP affects inflammation and immune response by regulating the TLR4/NF-κB signalling pathway, which has potential value for the treatment of IC.

In discussing the results of this study, some limitations should also be noted. First, due to funding constraints, this study lacked a comparison of inflammatory factors related to cystitis, and histological staining was not quantitatively analysed. In addition, although sequencing results showed that multiple proteins were involved in the protective effect of PRP on IC, the study only selected TLR4 for subsequent analysis, without conducting in-depth analysis of other proteins involved in the protective effect. We will supplement this in future research. In addition, this study only uses animal models, and more research support is needed for clinical transformation. Second, the mechanism of the TLR4/NF-κB signalling pathway needs to be further explored. Therefore, future research can be combined with clinical trials to further verify the effect and mechanism of PRP in IC treatment, with more related research conducted to provide new ideas and methods for IC treatment.

## Conclusion

In conclusion, the results of this study provide experimental evidence for the application of PRP in the treatment of IC and reveal the potential mechanism by which PRP improves bladder inflammation damage through the modulation of the TLR4/NF-κB signalling pathway. These findings offer valuable insights for the development of new treatment strategies for IC and may promote the research and development of more effective therapies. Future studies should focus on exploring the optimal dosage, treatment duration and long-term effects of PRP, as well as its application prospects in patients with IC. The study provides scientific evidence for the new treatment strategy of IC and provides a reference for future clinical application.

## Data Availability

All data generated or analysed during this study are included in this published article.

## References

[1] Li J, Yi X, Ai J. Broaden horizons: the advancement of interstitial cystitis/bladder pain syndrome. Int J Mol Sci. 2022;23(23):14594. 36498919 10.3390/ijms232314594 PMC9736130

[2] Su F, Zhang W, Meng L, et al. Multimodal single-cell analyses outline the immune microenvironment and therapeutic effectors of interstitial cystitis/bladder pain syndrome. Adv Sci (Weinh). 2022;9(18):e2106063. 35470584 10.1002/advs.202106063 PMC9218658

[3] Fang J, Wang X, Jiang W, et al. Platelet-rich plasma therapy in the treatment of diseases associated with orthopedic injuries. Tissue Eng Part B Rev. 2020;26(6):571–85. 32380937 10.1089/ten.teb.2019.0292 PMC9208862

[4] O’Connell B, Wragg NM, Wilson SL. The use of PRP injections in the management of knee osteoarthritis. Cell Tissue Res. 2019;376:143–52. 30758709 10.1007/s00441-019-02996-x

[5] Dickinson M, Wilson S. A critical review of regenerative therapies for shoulder rotator cuff injurie. SN Compr Clin Med. 2019;1(3):205–14.

[6] Alsousou J, Thompson M, Hulley P, et al. The biology of platelet-rich plasma and its application in trauma and orthopaedic surgery: a review of the literature. J Bone Joint Surg Br. 2009;91-B(8):987–96. 10.1302/0301-620X.91B8.22546 19651823

[7] Everts P, Onishi K, Jayaram P, Lana JF, Mautner K. Platelet-rich plasma: new performance understandings and therapeutic considerations in 2020. Int J Mol Sci. 2020;21(20):7794. 33096812 10.3390/ijms21207794 PMC7589810

[8] Yifan Yang, Zhifang Zhong, Yiming Liu, et al. Progress in the application of platelet rich plasma in the treatment of neuropathic pain. Chin J Pain Med. 2024;30(01):19–27.

[9] Cong P, Jing L, Xiaopeng T, et al. Research progress on the application of platelet rich plasma in horse sports system injury. Chin J Anim Husb Vet Med. 2024;51(04):1784–91.

[10] Min Z, Fei’e Z. Research progress on autologous platelet rich plasma for the treatment of discogenic lower back pain. Chin J Pain Med. 2024;30(02):131–6.

[11] Dong Q, Weijing Q, Jieshi H, et al. The effect of autologous platelet rich plasma combined with ozone treatment on ankle traumatic arthritis caused by military training and its impact on bone metabolism indicators. Clin Med Res Pract. 2024;9(08):27–30.

[12] Olona A, Hateley C, Muralidharan S, et al. Sphingolipid metabolism during toll-like receptor 4 (TLR4)-mediated macrophage activation. Br J Pharmacol. 2021;178(23):4575–87. 34363204 10.1111/bph.15642

[13] Kovler ML, Gonzalez Salazar AJ, et al. Toll-like receptor 4-mediated enteric glia loss is critical for the development of necrotizing enterocolitis. Sci Transl Med. 2021;13(612):eabg3459. 34550727 10.1126/scitranslmed.abg3459 PMC8859973

[14] Rosen JM, Klumpp DJ. Mechanisms of pain from urinary tract infection. Int J Urol. 2014;21(Suppl 1(0.1)):26–32. 24807489 10.1111/iju.12309 PMC4552327

[15] de Oliveira MG, Mónica FZ, Calmasini FB, et al. Deletion or pharmacological blockade of TLR4 confers protection against cyclophosphamide-induced mouse cystitis. Am J Physiol Renal Physiol. 2018;315(3):F460-8. 29717937 10.1152/ajprenal.00100.2018

[16] Engin S, Barut EN, Yaşar YK, et al. Trimetazidine attenuates cyclophosphamide-induced cystitis by inhibiting TLR4-mediated NFκB signaling in mice. Life Sci. 2022;301:120590. 35504331 10.1016/j.lfs.2022.120590

[17] Nagae M, Ikeda T, Mikami Y, et al. Intervertebral disc regeneration using platelet-rich plasma and biodegradable gelatin hydrogel microspheres. Tissue Eng. 2007;13:147–58. 17518588 10.1089/ten.2006.0042

[18] Li H, Zhang F, Pan Y, et al. Platelet-rich plasma versus autologous blood versus steroid injection in lateral epicondylitis: systematic review and network meta-analysis. J Hand Surg. 2018;43(6):14. 10.1007/s10195-015-0376-5 PMC4882297 26362783

[19] Reszec J, Hermanowicz A, Rutkowski R, et al. Expression of Toll-like receptors 2, 4 and nuclear factor κB in transitional cell carcinoma of the bladder. Int J Exp Pathol. 2020;101(4):8.

[20] Kim HR, Kim JW, Park YK, et al. Effect of platelet-rich plasma on ultraviolet b-induced skin wrinkles in nude mice. Arch Plast Surg. 2018;45(6):18. 10.1016/j.bjps.2010.08.014 20884308

[21] Zusso M, Lunardi V, Franceschini D, et al. Ciprofloxacin and levofloxacin attenuate microglia inflammatory response via TLR4/NF-kB pathway. J Neuroinflammation. 2019;16(1):148. 31319868 10.1186/s12974-019-1538-9 PMC6637517

[22] Jiang B, Wang D, Hu Y, et al. Serum amyloid A1 exacerbates hepatic steatosis via TLR4-mediated NF-κB signaling pathway. Mol Metab. 2022;59:101462. 35247611 10.1016/j.molmet.2022.101462 PMC8938331

[23] Ye Y, Jin T, Zhang X, et al. Meisoindigo protects against focal cerebral ischemia-reperfusion injury by inhibiting NLRP3 Inflammasome activation and regulating microglia/macrophage polarization via TLR4/NF-κB signaling pathway. Front Cell Neurosci. 2019;16(13):553. 10.3389/fncel.2019.00553 PMC6930809 31920554

[24] Guan, Y, Ruan, J, Tan, P, et al. Hesperidin alleviates endothelial cell inflammation and apoptosis of Kawasaki disease through inhibiting the TLR4/IĸBα/NF-ĸB pathway. CHEM-BIOL INTERACT. 2023; 411:111445. 10.1016/j.cbi.2025.111445 10.1016/j.cbi.2025.111445 39987982

[25] Chen Y, Hu Q, Muller G. Abscopal abscopal effect of high intensity focused ultrasound and checkpoint inhibition on prostate cancer-bearing mice. Photoacoustics. 2020;18(8):22.

